# The effect of ginger (*Zingiber officinale* L.) liquid extract on growth, immune response, antioxidant defence mechanism, and general health of Holstein calves

**DOI:** 10.1007/s11250-024-03972-6

**Published:** 2024-04-11

**Authors:** Serkan Özkaya, Fahruddin Pigamov, Sabri Erbaş, Murat Mutlucan, Ulaş Evren Arın, Elif Rabia Şanlı

**Affiliations:** 1 Department of Animal Science, Agriculture Faculty, Isparta University of Applied Sciences, Isparta, Turkey; 2 Department of Field Crop, Agriculture Faculty, Isparta University of Applied Sciences, Isparta, Turkey; 3 Rose and Aromatic Plants Implementation and Research Centre, Isparta University of Applied Sciences, Isparta, Turkey; 4 Department of Anesthesia, Isparta Vocational School of Health Services, Suleyman Demirel University, Isparta, Turkey

**Keywords:** Ginger, Growth, Immunity, Oxidative stress, Health, Calves

## Abstract

This study was carried out to examine the effects of ginger liquid extract (GLE) on the growth, immune response, antioxidative defence mechanism, and general health of Holstein calves. Sixteen calves (4-d old) were included in the experiment and randomly assigned to groups, and they were fed whole milk containing GLE at a rate of 0, 0.50, 0.72, and 1% of the milk amount consumed. Calves consuming 1% GLE were weaned at an earlier age and gained better body weight (BW) compared to the other groups. The group fed with 0.50% GLE consumed less daily starter than the other groups. The administration of GLE resulted in a non-significant decrease in fecal score (FS), the number of days with diarrhea (DDN), and illness (IDN) among the calves. Notably, the 1% GLE exhibited a significant inhibitory effect on the growth of *E. coli*, while its effect on the growth of other pathogenic bacteria was not statistically significant. Despite the non-significant reduction in malondialdehyde (MDA), total oxidative status (TOS), and oxidative stress index (OSI) values, the 1% GLE demonstrated support for antioxidative defence mechanism and immune response. The results indicated that 1% GLE can be effective in promoting the health of calves by enhancing their immune response and antioxidant capacity. This suggests that incorporating 1% GLE into their overall well-being, potentially leading to improved health outcomes and performance in calf rearing operations.

## Introduction

Ginger, which has been used medicinally in China and India for many years, is one of the plants used as an alternative to antibiotics. Ginger has various pharmacological properties, primary antimicrobial and anticancer (Da Silveira Vasconcelos et al. 2019). Used as a spice and flavouring, ginger has antioxidant and antihypercholesterolemic activity. In addition, ginger, which is rich in essential oils and minerals, can be used to increase productivity in livestock. The stimulating effect of the bioactive components of ginger on gastric secretion and enzymes positively affects feed efficiency and nutrient utilization (Ogbuewu and Mbajiongu 2020). In addition, it positively affects lipid digestion and absorption by increasing the synthesis of bile acids in the liver and their excretion in bile (Prakash and Srinivasan 2012).

It has been reported that ginger root powder affects feed consumption, milk production and milk quality without adversely affecting the health of cows (Al-dain and Jerjeis 2015). The supplementation of the olebiotic flavour obtained from ginger to the calf starter does not affect the milk and feed consumption, body weight gain, feed conversion ratio, plasma glucose value, β-hydroxybutyrate, blood urea nitrogen and creatine values of the calves (Davarmanesh et al. 2015).

It seems that the studies on ginger liquid extract’s effects, particularly concerning calves’ growth performance, feed utilization, intestinal bacterial flora, immune response, and oxidative stress, are scarce. Given this knowledge gap, the aim of this study was to investigate whether ginger extract could potentially contribute to the health rearing of calves. By testing this hypothesis, the research seeks to fill the existing void in understanding the potential benefits and implications of ginger liquid extract in calf husbandry practices.

## Materials and methods

### Ethics committee approval

The study was approved by the Animal Experiments Local Ethics Committee of Isparta University of Applied Sciences on 27.05.2021 with protocol number 001.

### Animal and feed material

Newborn Holstein calves (*N* = 16, 4 in each group) in Isparta University of Applied Sciences Agriculture Faculty Education, Research and Application Farm Dairy cattle unit were used in the study. The power analysis method used to determine the sample size of calves in the study. The analysis considered literature values related to the number of days with diarrhea, where the highest average was 4.30, the lowest average was 1.0 and the standard deviation was 0.40, and with a targeted power 95%, it was determined that there should be 4 calves in each group.

Commercially available calf starter containing 18.17% crude protein and 2848.90 kcal/kg metabolic energy was used in the study. The calf starter was received to the groups as standard throughout the study. During the suckling period, the calves were not given barley straw and alfalfa hay.

Mixed milk from the cows on the farm was used to feed the calves (3.3% CP, 3.4% fat, and 5.8% lactose).

### Experimental management

Calves were separated from their dams 45 min after birth and were fed with colostrum for the first 3 days. BW and body measurements (BMs) were taken at 4-day-old and they were randomly divided into groups (BMs: Body length, wither height, hip height, body depth, chest girth). The calves received 2 lt of milk in a bottle as 2 meals in the morning and evening. GLE was determined as the percentage of milk consumed by calves and mixed into their milk. GLE was mixed into the milk of the calves at varying rate of 0, 0.50, 0.72, and 1% as per the protocol outlined by Kishk and El-Sheshetawy (2010). Groups were formed as control (0% GLE), T1 (0.50% GLE), T2 (0.72% GLE), and T3 (1% GLE). The calves were weaned when they consumed 0.800 kg of starter for 3 consecutive days.

### Ginger liquid extract and phenolic substance amount of the ginger liquid extract

The ginger powder obtained from the spice trader was mixed with distilled water at a concentration of 1%. It was kept at 22 ± 2 ℃ for 18 h. Then, a liquid extract was obtained by filtration (Kishk and El-Sheshetawy 2010).

Phenolic content was determined by the spectrophotometric method (Eberhardt et al. 2000).

### Antioxidant activity and iron-reducing power of the ginger liquid extract

According to Shimada et al. (1992), antioxidant activity was determined by the DPPH (1,1-diphenyl-2-picryl-hydrazil) method and free radical scavenging activity was calculated using the following formula:

$$\begin{aligned}&\text{Antiradical}\;\text{activity} \left(\%\right) = \left(\right(\text{absorbance}\;\text{value}\;\text{of}\;\text{control} \\&\;\;- \text{eg}\;\text{absorbance}\;\text{value})/(\text{absorbance}\;\text{value}\;\text{of}\;\text{control}\left)\right) \times 100 \end{aligned}$$

The iron-reducing power of GLE was determined according to the method specified by Oyaizu (1986). The obtained value was compared with synthetic materials such as butylated hydroxyanisole (BHA), butylated hydroxytoluene (BHT) and 6-hydroxy-2, 5, 7, 8-tetramethylchroman-2-carboxylic acid (Trolox).

### Growth performance and health

BW and BMs of the calves were recorded at the beginning of the experiment and weekly thereafter, as stated by Ozkaya and Bozkurt (2009). The amount of starter consumed by the calves was recorded daily using an electronic scale with 1 g precision (TESS, Coymak Tartı LTD, Turkiye).

The fecal score of the calves, fecal samples and bacterial count in the feces were taken and counted as stated by Ozkaya et al. (2018).

Blood samples were taken from the jugular veins of each calf in the groups at the beginning of the trial and weaning. Immunoglobulins (Immunoglobulin-A (IgA), M (IgM), and G (IgG)), antioxidative enzyme activity (Total antioxidant status (TAS), catalase (CAT), superoxide dismutase (SOD), glutathione peroxidase (GPx)) and oxidative stress markers (Total oxidant status (TOS), Malondialdehyde (MDA)) were determined. Immunoglobulins, antioxidative enzyme activity and oxidative stress markers were determined by Baran Medical (Ankara, Turkiye) by spectrophotometric and Elisa methods.

The number of days with diarrhea and disease of the calves was recorded daily. Calves with a fecal score ≥ 3 for two consecutive days were recorded as having diarrhea (Ozkaya et al. 2018).

### Statistically analyses

The data obtained in the study were analyzed with the GLM procedure of the variance analysis technique. The differences among the groups were examined with the Tukey test. Minitab 20 package program was used for statistical analysis (Minitab 20 v1.10, Minitab Ltd. UK).

## Results

### Total phenolics, antiradical activity, and iron-reducing power

The average extract yield of the powder obtained from ginger root is 3.21% and the mean of its total phenolics is 5.23 mg/g GEA/ml. In an in vitro study to determine the antiradical activity of ginger powder, it showed antiradical activity like synthetic antioxidants. The iron-reducing power of ginger powder appears to be high in synthetic antioxidants.

### Performance of calves with ginger liquid extract

Calves fed 1% GLE had statistically the highest BW at both 4wk, 6wk and 8wk. Therefore, total and daily body weight gains at early weaning age were higher than the other groups (*P* < 0.05) (Table 1). In addition, wither height of calves was statistically highest in calves fed 0.72 GLE at 8 weeks compared to the other groups.

**Table 1:** The effect of ginger liquid extract on live weight and body measurements

	4 wk	Weaning age	8 wk	Daily Gain	Total Gain
	Mean ± S.E.	Mean ± S.E.	Mean ± S.E.	Mean ± S.E.	Mean ± S.E.
	*Live weight, kg*				
C	50.26 ± 2.68^a^	61.56 ± 3.61	71.29 ± 1.69^ab^	0.506 ± 0.02^ab^	29.87 ± 1.30^ab^
T1	45.59 ± 4.80^b^	57.21 ± 3.82	63.32 ± 6.93^b^	0.371 ± 0.05^b^	21.90 ± 2.68^b^
T2	48.87 ± 0.73^ab^	56.94 ± 1.69	67.91 ± 0.73^ab^	0.449 ± 0.02^ab^	26.50 ± 1.01^ab^
T3	50.45 ± 3.53^a^	55.29 ± 3.67	74.82 ± 3.49^a^	0.566 ± 0.05^a^	33.40 ± 2.95^a^
P	0.04	0.12	0.04	0.04	0.04
	*Body length, cm*				
C	76.50 ± 1.04	81.67 ± 1.33	84.67 ± 1.30	0.20 ± 0.01	13.17 ± 1.01
T1	74.17 ± 2.35	78.67 ± 1.86	81.00 ± 3.06	0.18 ± 0.02	11.83 ± 1.74
T2	77.83 ± 0.44	81.17 ± 0.60	84.67 ± 0.88	0.21 ± 0.01	12.83 ± 1.17
T3	72.50 ± 2.02	74.67 ± 2.42	81.67 ± 2.59	0.26 ± 0.04	16.50 ± 0.76
P	0.31	0.42	0.30	0.22	0.30
	*Wither height, cm*				
C	81.40 ± 2.20	85.86 ± 2.02^ab^	86.48 ± 2.03	0.14 ± 0.01	8.48 ± 0.33
T1	81.80 ± 2.33	84.96 ± 1.53^ab^	86.25 ± 2.09	0.14 ± 0.01	8.25 ± 0.67
T2	82.86 ± 0.88	85.82 ± 0.87^a^	88.06 ± 1.01	0.17 ± 0.01	10.07 ± 0.29
T3	82.11 ± 2.02	82.87 ± 2.02^b^	87.54 ± 2.31	0.15 ± 0.02	9.54 ± 1.20
P	0.37	0.04	0.35	0.29	0.35
	*Hip height, cm*				
C	84.27 ± 2.20	87.11 ± 1.96	88.77 ± 2.02	0.14 ± 0.01	9.10 ± 0.17
T1	83.62 ± 2.52	87.04 ± 1.80	88.22 ± 2.33	0.15 ± 0.01	8.55 ± 0.88
T2	85.08 ± 1.17	87.55 ± 0.58	89.37 ± 0.73	0.17 ± 0.02	9.71 ± 0.29
T3	84.27 ± 1.86	85.46 ± 2.35	89.98 ± 1.88	0.17 ± 0.02	10.31 ± 1.20
P	0.36	0.25	0.41	0.23	0.41
	*Body depth, cm*				
C	31.78 ± 1.01	33.61 ± 1.09	34.07 ± 1.09^b^	0.09 ± 0.01	4.40 ± 0.44^b^
T1	31.89 ± 1.20	34.39 ± 0.93	35.10 ± 1.26^ab^	0.11 ± 0.01	5.43 ± 0.29^ab^
T2	32.64 ± 0.83	34.64 ± 0.60	36.59 ± 0.60^a^	0.15 ± 0.02	6.92 ± 0.67^a^
T3	32.19 ± 0.50	32.86 ± 0.73	35.75 ± 0.50^ab^	0.18 ± 0.04	6.08 ± 0.44^ab^
P	0.75	0.12	0.04	0.06	0.04
	*Chest girth, cm*				
C	83.13 ± 3.24	89.87 ± 2.19	92.37 ± 2.32	0.32 ± 0.03	15.33 ± 1.17
T1	80.48 ± 2.96	86.95 ± 0.76	85.79 ± 2.60	0.17 ± 0.09	8.75 ± 3.32
T2	82.14 ± 1.61	88.65 ± 2.29	89.78 ± 3.48	0.25 ± 0.08	12.74 ± 3.21
T3	82.92 ± 1.20	84.21 ± 1.33	91.89 ± 0.58	0.45 ± 0.07	14.85 ± 0.88
P	0.08	0.08	0.24	0.06	0.24

^ab^defines the difference between column

### General health status of calves

Calves consuming GLE mixed milk showed a nonsignificant decrease in fecal scores, in the incidence of diarrhea and respiratory tract diseases.

During the suckling period, no significant differences were observed among the rectal temperature averages of the calves (Table 2).

No significant difference was found between the starter intake ages of the calves (Table 2). Total feed consumption decreased insignificantly in groups receiving 0.50 and 1% GLE (Table 2). However, daily feed consumption increased significantly (*P* < 0.05) in groups receiving 0.72 and 1% GLE.

The GLE did not have a significant effect on the feed conversion ratio of the calves (Table 2).

**Table 2:** The effect of ginger liquid extract on general health status, and feed intake of calves

	C	T1	T2	T3	P
	Mean ± S.E.	Mean ± S.E.	Mean ± S.E.	Mean ± S.E.	
Weaning age, d*	49.91 ± 2.08^ab^	51.50 ± 3.67^a^	45.87 ± 2.52^ab^	35.39 ± 4.04^b^	0.04
Illness day	2.00 ± 2.00	1.00 ± 1.00	0.67 ± 0.67	0.67 ± 0.67	0.85
Diarrhea day	4.33 ± 0.33	3.67 ± 2.03	3.33 ± 1.76	2.67 ± 1.33	0.89
Starter consumption age, d	4.67 ± 0.67	4.00 ± 0.00	4.67 ± 0.67	7.67 ± 2.33	0.24
Fecal scour	1.50 ± 0.05	1.34 ± 0.09	1.33 ± 0.06	1.31 ± 0.04	0.19
Rectal temperature, ℃	38.62 ± 0.05	38.52 ± 0.13	38.49 ± 0.12	38.83 ± 0.06	0.13
TSC, kg	16.57 ± 2.12	13.23 ± 1.73	16.57 ± 1.32	12.53 ± 1.33	0.25
DSC, kg*	0.35 ± 0.03^ab^	0.27 ± 0.02^b^	0.40 ± 0.03^a^	0.38 ± 0.01^a^	0.02
FCR	0.69 ± 0.06	0.77 ± 0.14	0.87 ± 0.03	0.69 ± 0.08	0.42

TSC: Total starter consumption, DSC: Daily starter consumption, FCR: Feed conversion ratio, ^ab^defines the difference between lines

GLE suppressed the growth of pathogenic bacteria in the intestinal flora, but this suppression was not significant in pathogenic and non-pathogenic bacteria other than *E. coli* (Table 3).

**Table 3:** The effect of ginger liquid extract on fecal microorganism counts

	C	T1	T2	T3	P
	Mean ± S.E.	Mean ± S.E.	Mean ± S.E.	Mean ± S.E.	
*Yeast + Mold*	1.43 ± 1.43	0.00 ± 0.00	0.00 ± 0.00	0.00 ± 0.00	0.44
*Enterobacteriaceae*	6.47 ± 0.16	6.16 ± 0.209	4.09 ± 2.05	4.03 ± 2.02	0.52
*Coliforms*	6.69 ± 0.12	6.37 ± 0.06	6.26 ± 0.14	4.10 ± 2.05	0.33
E. coli*	6.52 ± 0.04^a^	6.20 ± 0.10^a^	6.10 ± 0.10^a^	2.10 ± 2.10^b^	0.01
*Staphylococcus*	2.20 ± 2.20	2.03 ± 2.03	2.00 ± 2.00	0.00 ± 0.00	0.80
*Salmonella*	4.27 ± 2.14	4.22 ± 2.11	2.04 ± 2.04	2.00 ± 2.00	0.77
*Lactic acid*	6.95 ± 0.05	6.69 ± 0.21	6.51 ± 0.30	6.52 ± 0.26	0.51

^ab^defines the difference between lines

The TOS value tended to decrease with GLE, MDA value decreased insignificantly, while OSI value decreased significantly (*P* < 0.05) (Table 4). A non-significant increase in IgA and IgM values was observed with the GLE, however, the GLE significantly increased the IgG value (*P* < 0.05) (Table 4).

**Table 4:** The effect of ginger liquid extract on oxidative stress, antioxidative stress mechanism and immune system

		C	T1	T2	T3	P
		Mean ± S.E.	Mean ± S.E.	Mean ± S.E.	Mean ± S.E.	
TAS, mmol/L	Int	1.08 ± 0.07	0.89 ± 0.07	1.09 ± 0.01	0.96 ± 0.06	0.62
	Fnl	0.95 ± 0.14	0.84 ± 0.12	1.08 ± 0.06	1.03 ± 0.09	
TOS, µmol/L	Int	5.49 ± 1.00	4.86 ± 2.54	2.54 ± 1.15	4.13 ± 0.33	0.07
	Fnl	1.17 ± 0.38	0.35 ± 0.03	3.61 ± 2.91	2.76 ± 2.19	
OSI*	Int	0.52 ± 0.12^a^	0.55 ± 0.27^a^	0.23 ± 0.10^b^	0.44 ± 0.07^a^	0.04
	Fnl	014 ± 0.06^b^	0.04 ± 0.01^b^	0.31 ± 0.25^a^	0.14 ± 0.19^b^	
CAT, U/L	Int	189.00 ± 13.4	219.60 ± 88.90	88.10 ± 20.20	133.30 ± 20.00	0.13
	Fnl	120.90 ± 21.50	143.40 ± 52.10	99.80 ± 40.00	87.10 ± 25.30	
SOD, U/ml	Int	273.90 ± 12.80	272.90 ± 14.30	232.30 ± 25.30	293.00 ± 26.70	0.37
	Fnl	269.90 ± 14.60	346.90 ± 32.30	238.50 ± 51.00	294.70 ± 42.10	
GPX, U/L	Int	699.70 ± 68.00	441 ± 40.40	749.00 ± 183.00	602.00 ± 139.00	0.28
	Fnl	603.70 ± 27.70	528.00 ± 15.60	518.00 ± 56.30	527.30 ± 23.100	
MDA, mmol/L	Int	7.36 ± 0.83	7.11 ± 1.39	4.57 ± 0.81	5.41 ± 0.89	0.42
	Fnl	3.32 ± 0.40	3.40 ± 0.80	9.28 ± 6.28	2.69 ± 0.29	
IgA, mg/dl	Int	1.84 ± 0.27	3.79 ± 1.98	0.25 ± 0.04	1.40 ± 0.70	0.33
	Fnl	1.33 ± 1.22	2.29 ± 2.21	0.37 ± 0.24	0.20 ± 0.01	
IgM, mg/dl	Int	7.54 ± 3.34	7.55 ± 1.85	8.04 ± 2.31	6.64 ± 1.46	0.78
	Fnl	3.90 ± 2.23	7.73 ± 2.75	10.05 ± 3.11	5.22 ± 2.00	
IgG, µg/ml*	Int	71.65 ± 7.03^a^	73.06 ± 4.22^a^	54.59 ± 4.78^b^	67.67 ± 3.18^ab^	0.00
	Fnl	90.90 ± 10.2^a^	83.04 ± 9.35^ab^	71.90 ± 6.26^b^	79.87 ± 4.20^ab^	

^ab^Defines the difference between lines

## Discussion

### Total phenolics, antiradical activity, and iron-reducing power

The phenolic compounds of the different extracts of ginger plant grown in two different regions were compared and it was reported that the phenolic substance of the methanol extract were 1183.813 and 1022.409 mg GAE/100 g, and ethanol extract was obtained as 748.865 and 670.152 mg GAE/100 g. At the same time, they reported that the antiradical activity of the acetone extract was high and the methanol extract was low. This difference may be due to different internal and external factors such as soil type and conditions in which it is grown, maturity and harvesting conditions of the ginger plant, as well as the difference in the extraction method (Ezez and Tefera 2021). The reason why the amount of phenolic substance obtained in the study was different from the previous ones may be due to the different methods of obtaining the ginger extract used and other conditions.

### Performance of calves with ginger liquid extract

Medicinal aromatic plants and their extracts, by stimulating the digestive system, enhancing enzyme release, and promoting gastric and intestinal motility, increase feed consumption. This phenomenon, supported by (Ozkaya et al. 2018; Isik and Ozkaya 2021), enables the early weanibg of calvees. Therefore, the GLE facilitates early weaning.

Plants by increasing the concentration of total volatile fatty acids in the rumen, enhance feed digestibility (Ozkaya 2020; Ozkaya et al. 2020), improve intestinal flora and positively effect on BW gain (Ozkaya et al. 2018; Isik and Ozkaya 2021). Several researchers have reported significant enhancements in calf BW gain through herbal extract supplementation (Ghosh et al. 2010). The findings from these studies indicate that the most favorable BW gain occurs with 1% GLE. Conversely, some studies suggest that the BW gain remains unaffected (Ozkaya et al. 2018; Isik and Ozkaya 2021).

The supplementation of aromatic plants and their extracts does not affect the BMs of calves (Ozkaya et al. 2018; Isik and Ozkaya 2021). GLE did not affect other BMs of calves except body depth measurement.

### General health status of calves

Aromatic oils and extracts support the immunity and reduced the incidence of disease in calves (Sajjadi et al. 2014; Zeng et al., 2014 2015; Ozkaya et al. 2018; Isik and Ozkaya 2021). Indeed, GLE showed a non-significant reduction in the NID in calves.

Herbal extracts suppress the growth of pathogenic bacteria in the intestinal flora, leading improvements in the intestinal system and a reducetion in diarrhea incidence. Additionally, herbal plant oil and extracts significantly reduce the fecal score (Ghosh et al. 2010, 2011; Ozkaya et al. 2018; Isik and Ozkaya 2021). However, GLE insignificantly decreased the NDD and fecal scores of the calves.

No significant differences were found in the rectal temperatures of the calves. The mean values remained within the normal limits (37.8–39.4 ℃) for calves (Donald 2005). Güneş (2008) reported that rectal temperature surpasses 39.5 ℃ in case of both respiratory tract and digestive system diseases.

The appetizing properties of medicinal aromatic plants and their extracts stimulate digestion and increase feed consumption by promoting gastric and intestinal motility by increasing the release of enzymes. Increased feed consumption allows animals to wean at an early age (Ozkaya et al. 2018; Isik and Ozkaya 2021). GLE (0.50%) enabled calves to start starter consumption at an early age, while the 1% GLE delayed starter consumption. In their study with oregano oil, it was reported by Tapki et al. (2020) that calves started consuming starter at an early age.

Plant extracts improve the digestion of feed by increasing saliva, bile and enzyme activities. However, as a result of the suppression of pathogenic bacteria in the intestine, it increases the ability of epithelial cells to regenerate the vili and significantly increases feed consumption by increasing the digestive and absorption capacity of the intestine (Ghosh et al. 2010; 2011), Similarly, it has been reported that the supplementation of Juniper aromatic water increases the feed consumption of calves (Isik and Ozkaya 2021). However, study is reporting that plant extracts do not affect feed consumption (Ozkaya et al. 2018). This difference in the results plot may be caused by systemic losses in the mucus secretion of herbal extracts (Jamroz et al. 2006).

The GLE did not affect the feed conversion ratio. The 1% GLE-applied group had the same feed conversion ratio as the control group, however, the other groups did not improve their feed conversion ratio. Hassan et al. (2020) reported that *Corymbia citriodora* leaf extract did not affect feed conversion, while Tapki et al. (2020) reported that oregano essential oil significantly improved it.

The GLE led to a reduction in TOS and MDA values while concurrently enhancing the activity of antioxidant enzymes such as CAT, SOD, and GPx (Table 4). This underscores the role of GLE in bolstering the antioxidant defense mechanism and mitigating the effects of oxidative stress. Indeed, previous studies on poultry have reported similar findings, demonstrating that ginger powder supplementation decreased MDA levels and enhanced liver SOD activity, although no significant differences were observed in GPx and CAT enzyme activities (An et al. 2019; Aikpitanyi and Egweh 2020).

Furthermore, although a non-significant effect of GLE on IgA and IgM levels was observed (Table 4), a significant increase in IgG concentration was observed. This aligns with findings from previous research on sheep, where the addition of ginger powder and oil resulted in a significant elevation of IgG concentration. Such effects can be attributed to the beneficial alterations induced by medicinal aromatic plants in the duodenal mucosa, ultimately enhancing immune function (Bakr 2019).

In the investigation into the effects of GLE administration on the health of calves, it was noted that the inclusion of 1% GLE led to improvements in LW gain and overall body growth of the calves. Moreover, owing to its favorable effect on intestinal flora, it resulted in reduction in the occurrence of digestive and respiratory ailments among the calves. By bolstering the immune system and antioxidative defense mechanism, it facilitated the healthy rearing of the calves. Consequently, it was concluded that GLE, being easy to administer, could serve as a viable alternative feed additive for calf rearing practices.

## Data Availability

Datasets obtained and/or analyzed during the current study are available from corresponding after upon reasonable request.
